# Phyllanthi Tannin Loaded Solid Lipid Nanoparticles for Lung Cancer Therapy: Preparation, Characterization, Pharmacodynamics and Safety Evaluation

**DOI:** 10.3390/molecules28217399

**Published:** 2023-11-02

**Authors:** Baojin Wang, Kai Wu, Runping Liu, Ya Huang, Zihao Chang, Ye Gao, Yuqi Liu, Hongjiao Chen, Zhaohui Wang, Yitong Cui, Le Wang, Pengkai Ma, Lanzhen Zhang

**Affiliations:** School of Chinese Pharmacy, Beijing University of Chinese Medicine, Beijing 102488, China; wbj991102@163.com (B.W.); wukaiLX@163.com (K.W.); lrpscreen@live.com (R.L.); hya9993@163.com (Y.H.); changzh1995@126.com (Z.C.); gy_yeah1415926@163.com (Y.G.); 15201690695@163.com (Y.L.); 20230941462@bucm.edu.cn (H.C.); wangzhaohui0426@163.com (Z.W.); cuiyitong@126.com (Y.C.); wangle_0421@163.com (L.W.)

**Keywords:** *Phyllanthus emblica* L., anti-tumor, solid lipid nanoparticles, lung cancer, polyphenol, safety evaluation

## Abstract

The objective of the present study was to develop PTF-loaded solid lipid nanoparticles (PTF-SLNs) and investigate their efficacy in treating lung cancer. The PTF-SLNs were prepared by the thin film hydration method and verified by FTIR and TEM. Their physicochemical properties were characterized by particle size, polydispersity index (PDI), zeta potential, entrapment efficiency (EE), drug loading (DL), etc. Then, the pharmacodynamic studies of PTF-SLNs were performed on Lewis lung cancer cells and tumor-bearing mice. Finally, the safety studies were assessed by organ index, serum biochemical indicators, and histopathological changes. The PTF-SLNs were characterized by around 50 nm sphere nanoparticles, sustained ideal stability, and controlled drug release effects. The pharmacodynamic evaluation results showed that PTF-SLNs had stronger anti-tumor efficacy than PTF. An in vitro study revealed a more obvious cytotoxicity and apoptosis effect. The IC 50 values of PTF and PTF-SLNs were 67.43 μg/mL and 20.74 μg/mL, respectively. An in vivo study showed that the tumor inhibition rates of 2 g/kg PTF and 0.4 g/kg PTF-SLNs were 59.97% and 64.55%, respectively. The safety preliminary study indicated that PTF-SLNs improve the damage of PTF to normal organs to a certain extent. This study provides a nanoparticle delivery system with phenolic herbal extract to improve anti-tumor efficacy in lung cancer.

## 1. Introduction

Cancer is still the main cause of death in the world. Specifically, lung cancer has the highest mortality due to its high incidence rate and low survival rate [1]. At present, the conventional treatment methods for lung cancer are surgery, radiotherapy, chemotherapy, and targeted therapy. These methods have minimal benefits as they can lead to serious adverse reactions and the progression of the disease [2,3]. Although patients also receive treatment with standard chemotherapy drugs, their prognosis is relatively poor, with a survival rate of only 15% within 5 years [4]. Traditional chemotherapy has been eclipsed in cancer treatment owing to the emergence of cell resistance [5,6]. Thus, this is a very urgent search for safe and effective drugs to prevent lung cancer. However, advanced molecular medicine therapy is expensive and inconvenient for patients [7]. Natural herbal products play an extremely promising role in the whole course of cancer prevention and treatment [8].

*Phyllanthus emblica* L., a traditional Tibetan medicinal herb, has been used clinically in Tibet for thousands of years. Modern pharmacological research shows that its active extracts have anti-tumor, anti-inflammatory, antibacterial, antiviral, and other activities due to their rich content of tannins and polyphenols [9,10,11]. In addition, our previous studies demonstrated that phyllanthi tannin fraction (PTF) significantly induced apoptosis, inhibited the migration and invasion of human lung squamous carcinoma cells in vitro via MAPK/MMP pathways, and was also cytotoxic to Lewis lung cancer cells by inhibiting cell clone formation and inducing cell apoptosis [12]. However, the oral bioavailability and solubility of PTF were poor, so the oral administration dose was extremely high, which also led to other side effects such as indigestion. Therefore, we need to find reliable strategies to overcome the weaknesses related to PTF for further clinical application.

The nano-drug delivery system has been a promising approach to improving the properties of drugs in recent decades, including drug solubility, tumor-targeting ability, and drug toxicity reduction ability. Various nano-drug delivery systems have been investigated for tumoral drug delivery, such as polymeric nanoparticles, liposomes, micelles, solid lipid nanoparticles (SLNs), etc. [13]. Among these, SLNs are flexible nanocarriers with an average particle size of 10 to 1000 nm, used for drug delivery in almost all routes of administration, including ocular, parenteral, oral, and dermal. They have the ability to sustain drug delivery, consequently decreasing the frequency of administrations and improving therapeutic effectiveness. In addition, they can improve drug bioavailability and achieve targeting [14]. The methods for preparing SLNs are very mature and include the emulsion solvent diffusion method, the thin film hydration method, the high-pressure homogenization method, etc. SLNs have gained significant attention in delivering chemical drugs such as Paclitaxel, Adriamycin, and Doxorubicin owing to their ability to improve physical stability, their controlled release, low carrier toxicity, high drug load, and easy scale-up process [15]. Furthermore, SLNs are also increasingly being applied in herbal extracts, including flavonoid SLNs [16], as the advantage of carrying both hydrophilic and hydrophobic drugs has been proven in recent years. Hence, we chose SLNs as our nanocarrier for the delivery of PTF to treat lung cancer based on its multiple advantages.

In this study, we aim to formulate and evaluate PTF in SLNs using different solid lipid amounts and surfactant percentages. Furthermore, we characterized the physicochemical properties of PTF-SLNs, including mean particle size, PDI, zeta potential, entrapment efficiency (EE), drug loading (DL), and so on. In addition, to evaluate the therapeutic effect of PTF-SLNs, the pharmacodynamics and safety were investigated in in vitro and in vivo studies.

## 2. Results and Discussion

### 2.1. Optimization of PTF-SLNs

The SLNs drug delivery system is a colloidal aqueous dispersion composed of solidified lipids and surfactants [17,18,19]. The formulation of PTF-SLNs was investigated, as shown in Table 1 and Table 2. The GMS was selected as a solid lipid owing to the high DL of the prepared PTF-SLNs. Brij^®^58, as a nonionic surfactant class, was selected as the surfactant to form the stable PTF-SLNs suspension. In addition, Brij^®^58 has been proven to possess the distinguished effect of controlled drug release [20,21,22] and low toxicity for potential applications such as anti-tumor activity [23,24]. Moreover, single-factor results revealed that the optimal formula of PTF-SLNs was 3.75 mg GMS, 30 mg lecithin, 10 mg Brij^®^58, 10 mg PTF, and 10 mL purified water.

Freeze-drying is a reliable strategy that can remove water from the nanosuspension. The Brownian motion of nanoparticles is restricted, reducing the chance of particle aggregation upon storage and improving the long-term stability of nanodrugs. Cryoprotectants are generally recognized as requisite excipients to ensure that the quality of nanoparticles is maintained throughout the freeze-drying process [25]. The influence of cryoprotectants on the quality of PTF-SLNs was also investigated (Table 3). When the cryoprotectant contained 6% sucrose, the freeze-dried products possessed a favorable appearance, good re-solubility, and uniform particle size. This phenomenon may be attributed to the strong and powerful hydrogen bonding formed between sucrose and the surface of SLN, better protecting them during dehydration [25].

### 2.2. HPLC Analysis

**Figure 1:**
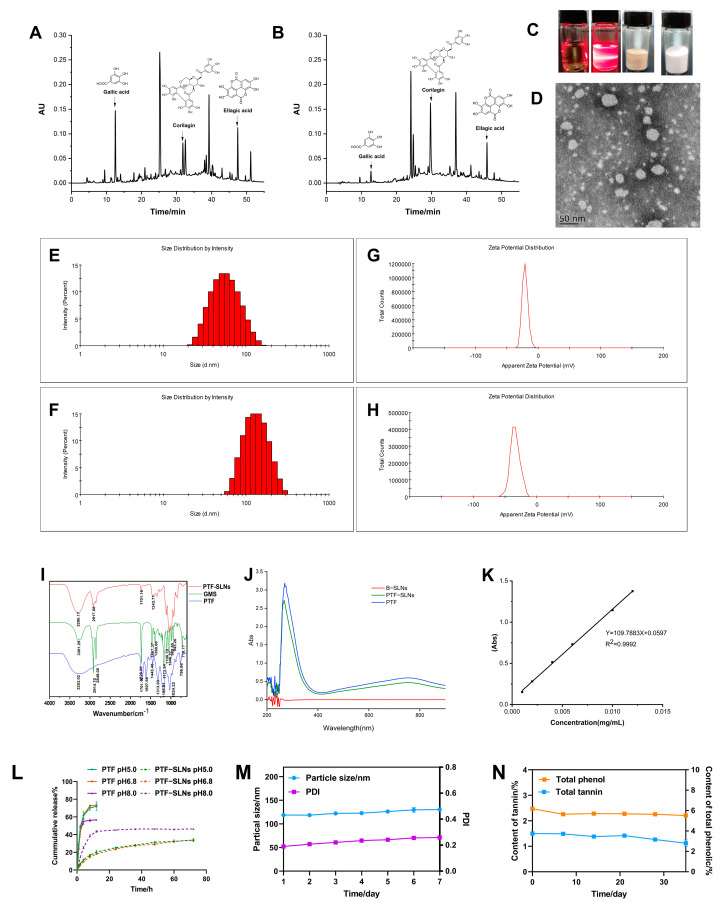
Characterization of PTF-SLNs. (**A**) The HPLC chromatogram of the PTF; (**B**) The HPLC chromatogram of the PTF-SLNs; (**C**) The picture of PTF solution, PTF-SLNs suspension, PTF-SLs freeze-dried sample, and B-SLNs freeze-dried sample; (**D**) TEM images of PTF-SLNs suspension; (**E**) The particle size distribution of PTF-SLNs suspension; (**F**) The particle size distribution of PTF-SLNs freeze-dried powder; (**G**) The zeta potential distribution of PTF-SLNs suspension; (**H**) The zeta potential distribution of PTF-SLNs freeze-dried powder; (**I**) IR spectroscopy of PTF-SLNs, GMS, and PTF; (**J**) UV scanning spectroscopy of B-SLNs, PTF-SLNs, and PTF; (**K**) Standard curve of gallic acid; (**L**) The in vitro drug release curve of PTF-SLNs and PTF in different pH mediums; (**M**) The particle size and PDI change curve within one week; (**N**) The TPC and TTC change curve within one month.

The PTF, an essential part of the water extract of *Phyllanthus emblica* L., has been found to possess various phenols and tannins. The anti-tumor components of PTF have been investigated, such as gallic acid, corilagin, ellagic acid, and so on [26]. The HPLC chromatogram of the PTF and PTF-SLNs (Figure 1A,B) showed the components share peaks, including gallic acid, corilagin, ellagic acid, etc. The results demonstrated that the active components of PTF were successfully loaded into SLNs, indicating that the method was reliable for preparing PTF-SLNs.

### 2.3. Characteristics of PTF-SLNs

The freeze-dried PTF-SLNs presented a loosely round cake-like powder that was easily re-dispersed in water, and the PTF-SLNs suspension was a translucent emulsion with light yellow opalescence (Figure 1C). The TEM observation confirmed its sphere morphology and good dispersibility (Figure 1D). In addition, the particle sizes of PTF-SLNs suspension and freeze-dried powder were 51.36 ± 0.51 nm and 118.9 ± 0.66 nm (Figure 1E,F), a negative zeta potential of −20.37 ± 0.76 mV and −33.57 ± 1.50 mV (Figure 1G,H), preventing aggregation and ensuring long-term stability. This may be due to the presence of long-chain fatty acids. The PDI of PTF-SLNs suspension and freeze-dried powder was 0.14 ± 0.01 and 0.21 ± 0.02, respectively, including a uniform distribution of PTF-SLNs, since PDI value is less than 0.3, which usually specifies homogeneous particle size distribution [27].

The IR spectroscopy is shown in Figure 1I. The characteristic peaks of PTF at 3253 cm^−1^, 1701 cm^−1^, 1607 cm^−1^, 1445 cm^−1^, and 1313 cm^−1^ were observed. The GMS spectroscopy displays five characteristic IR bands. The peaks were noted at about 2914 cm^−1^, 2849 cm^−1^, 1729 cm^−1^,1467 cm^−1^, and 1100 cm^−1^. The absorption peaks at 2914 cm^−1^ and 1100 cm^−1^ are the consequence of the antisymmetric stretching vibration of the −CH_2_ group, whereas the peak at 2849 cm^−1^ corresponds to the symmetric stretching vibration of the −CH_2_ group. The absorption peak at 1607 cm^−1^ (C=C) was shielded, and the transmittance of the absorption peak at 1701 cm^−1^ (C=O) was weakened after PTF formed PTF-SLNs, indicating the formation of hydrogen bonds due to interactions between PTF and GMS. The IR spectroscopy analysis verified the compatibility of the formulation’s constituent parts because of the unaltered peak locations. The IR results showed that there is no molecular incompatibility among the formulation components and that the components are compatible with one another. All of the results confirm the successful formation of the SLNs.

As seen in the ultraviolet absorption spectroscopy, the maximum absorption wavelength of both PTF-SLNs and PTF was 760 nm, and the B-SLNs had no UV absorption (Figure 1J), indicating that the nanocarriers will not interfere with TPC and TTC measurements. The standard curve of gallic acid (0.0010–0.012 mg/mL) was Y = 109.7883 X + 0.05973 (Figure 1K), and the TPC and TTC were measured based on gallic acid equivalents per gram of dry sample weight. The results for EE and DL were 73.83% and 5.00%, respectively. The TPC of PTF-SLNs was 42%. The results were satisfactory and could be attributed to the high encapsulation of the drug in the solid lipid and the stability brought by the surfactant.

The in vitro drug release curve is shown in Figure 1L. In general, the release of drugs from lipid-based colloidal systems is affected by several factors, including the temperature and nature of the release medium, drug load and drug position in the particles, the dimension and contour of the particles, the crystalline arrangement of the drug and the lipids of the matrix, the nature of the stabilizing agents and their organization around the particles, and the manufacturing method of the nanoparticles [28]. PTF prepared from PTF-SLNs can continuously release the drug for more than 72 h, and the drug release increased with increased pH. PTF was released explosively in 8 h, indicating that the PTF-SLNs had a noticeable sustained drug release effect and no initial burst release in different pH mediums. This continuous release behavior may be linked to the fact that the presence of hydrophobic long-chain fatty acids in the lipid-forming SLNs hinders drug release as a result of the essential for the drug to be released from the core-shell nanometer structure instead of being directly exposed to the release medium and, consequently, a more sustained release pattern is obtained [29].

The stable studies of freeze-dried PTF-SLNs showed that particle size and PDI remained negligible changes within a week, i.e., increased from 118.7 nm to 130.3 nm and 0.190 to 0.259, respectively (Figure 1M). The TPC and TTC were measured, and the results showed that the contents remained steady within one month and twenty days, respectively (Figure 1N).

### 2.4. Cytotoxicity Assay

**Figure 2:**
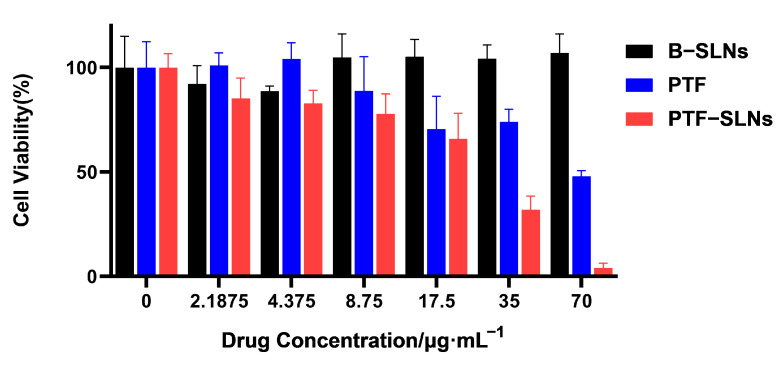
Cell viability was determined by CCK8 assay after the exposition of Lewis lung cancer cells to different concentrations of PTF, B-SLNs, and PTF-SLNs for 48 h.

The cell viability studies of PTF, B-SLNs, and PTF-SLNs were evaluated against Lewis lung cancer cells by the CCK-8 assay. The cell viability of B-SLNs was more than 90% at all concentrations. The PTF and PTF-SLNs showed concentration-dependent cytotoxicity, as presented in Figure 2. The calculated IC50 values for the free drug and PTF-SLNs were 67.43 μg/mL and 20.74 g/mL, respectively. It was observed that PTF-SLNs exhibited the strongest cytotoxicity effect against Lewis lung cancer cells compared with PTF. The results indicated that the PTF-SLNs increased the cytotoxic potential of PTF, and the nanocarrier provided high security.

### 2.5. Apoptosis Assay

**Figure 3:**
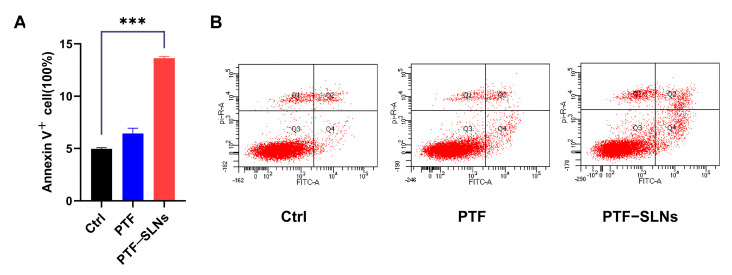
(**A**) Bar graphs show the percentage of specific cell populations by flow cytometric analysis. Compared with the control group, the PTF-SLNs group significantly increased Lewis lung cancer cell apoptosis (*** *p* < 0.001). (**B**) Representative dot plots of Lewis lung cancer cells after treatment with PTF and PTF-SLNs by the flow cytometric analysis.

The effect of inducing cell apoptosis in PTF-SLNs was also investigated by flow cytometry. The Lewis lung cancer cells were treated with PTF and PTF-SLNs after 48 h of incubation (Figure 3). Normal Lewis lung cancer cells served as control cells. The results have shown that 20 μg/mL PTF-SLNs enable 15% of cells to undergo apoptosis, and there was a substantial difference compared with the control group (*** *p* < 0.001). However, the PTF failed to induce significant apoptosis under the same conditions. The data indicated that PTF-SLNs increase the effect of PTF cell apoptosis.

### 2.6. Anti-Tumor Effect of PTF-SLNs

**Figure 4:**
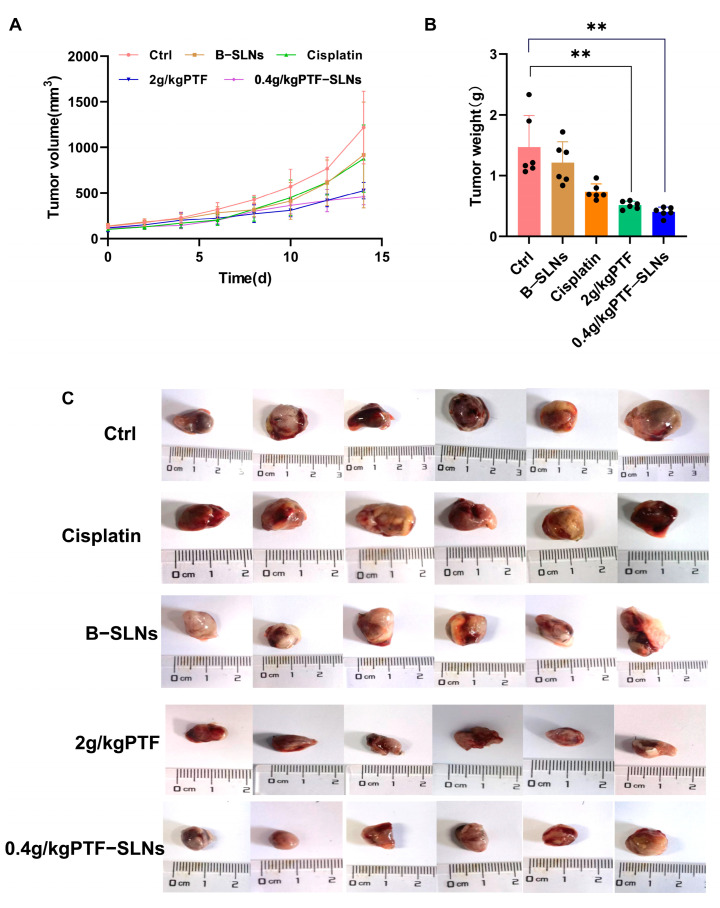
(**A**) The tumor growth curve of mice after oral administration of various formulations; (**B**) The tumor weight of each group, 2 g/kg PTF group and 0.4 g/kg PTF-SLNs group significantly decreased tumor weight (** *p* < 0.01) (*n* = 6); (**C**) Representative images of the tumor.

Our preliminary experiments found that the effective dosage of PTF was 2 g/kg, and the tumor inhibition rate was 54%. Therefore, the study used oral administration of 2 g/kg PTF as the control group and oral administration of 0.4 g/kg PTF-SLNs as the test group to inhibit tumor growth in Lewis lung cancer tumor-bearing mice. During the investigation, no obvious abnormality was found in the appearance, feeding, or activity state of the mice. In terms of tumor volume change, the tumor volume of the 2 g/kg PTF group and the 0.4 g/kg PTF-SLNs group mice was significantly lower than that of the model group (Figure 4A). The average tumor weight of the model group was 1.47 ± 0.71 g, indicating that the tumor growth was normal. The tumor weight of the PTF group and PTF-SLNs group showed a significant reduction compared with the model group (** *p* < 0.01) (Figure 4B). The tumor inhibition rate of PTF and PTF-SLNs was 59.97% and 64.55%, respectively (Table 4). In addition, the tumor regression of PTF-SLNs and PTF was stronger than cisplatin. The representative tumor images were shown in Figure 4C. The result indicated that PTF-SLNs performed a robust anti-tumor effect despite reducing the PTF dosage.

### 2.7. Safety Preliminary Study

#### 2.7.1. Organ Index

**Figure 5:**
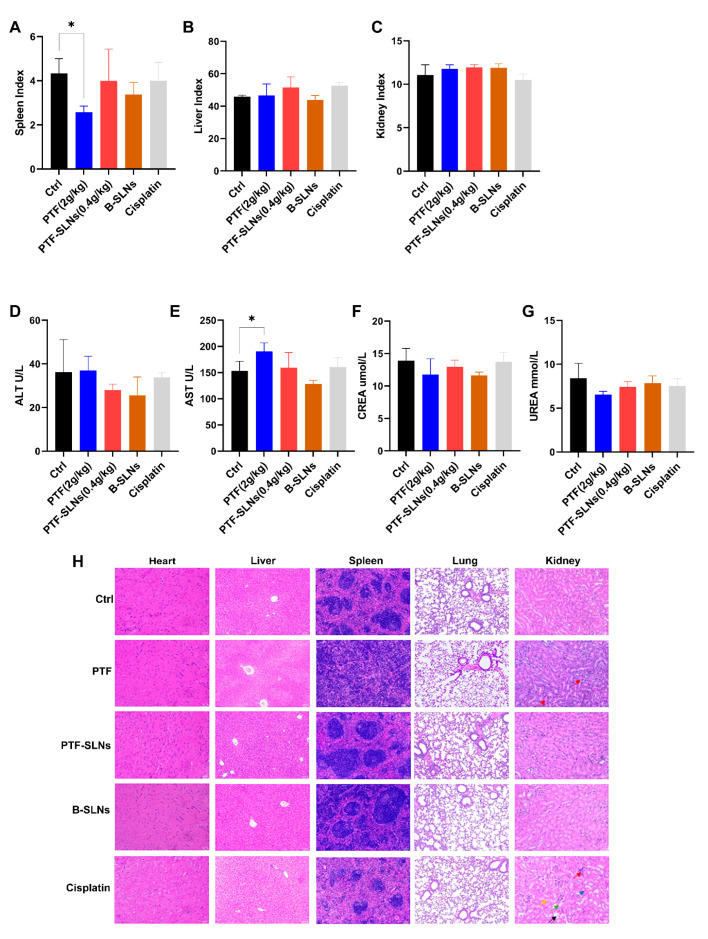
In in vivo safety testing, animals were treated with PTF, PTF-SLNs, B-SLNs, and cisplatin for 14 days. Organs were weighed to calculate the index of the spleen (**A**), liver (**B**), and kidney (**C**). The spleen index of the PTF administration group significantly decreased (* *p* < 0.05). The biochemical parameter was measured by an automatic biochemical analyzer. Blood samples were evaluated for the levels of ALT (**D**), AST (**E**), CREA (**F**), and UREA (**G**). The AST level of the PTF group showed a significantly elevated (* *p* < 0.05). (**H**) Effect of PTF, PTF-SLNs, B-SLNs, and cisplatin on tumor-bearing mice’s cardiac, hepatic, splenic, pulmonary, and renal histology.

The organ index was calculated by weighing to evaluate the influence of drugs on the spleen, liver, and kidney. The PTF-SLNs group had no significant impact on the spleen, liver, or kidneys (Figure 5A–C). Whereas the spleen index of the PTF administration group significantly decreased (* *p* < 0.05). Thus, the result indicated that PTF-SLNs could effectively reduce the toxicity of PTF to the spleen and proved that PTF-SLNs were secure in vivo.

#### 2.7.2. Measurement of AST, ALT, CREA, and UREA

AST, ALT, CREA, and UREA are cardinal liver and kidney function biomarkers. Mice in the PTF-SLNs group demonstrated relatively stable levels of the aforementioned biomarkers compared to the control group, and all parameter values in each group were within the normal range (Figure 5D–G). In contrast, the PTF group showed a significantly elevated (* *p* < 0.05) level of AST. The results indicated that oral administration of PTF to mice may cause a certain degree of liver damage. Furthermore, PTF-SLNs group mice showed that the above symptoms improved. In addition, PTF and PTF-SLNs did not negatively influence kidney function according to the parameters of UREA and CREA. To sum up, PTF-SLNs had higher safety in vivo than PTF.

#### 2.7.3. Histopathological Examination

Figure 5H represents the results of the viscus histopathology examination. The positive drug cisplatin group showed that severe kidney and liver injuries occurred, which were characterized by apparent necrosis of renal tubules, epithelial cell shedding (green arrow), cystic expansion of multiple tubules, eosinophilic material in some tubular lumens (black arrow), interstitial edema with inflammatory cell infiltration (red arrow), mild atrophy of renal tubule lumen, narrowing or disappearance of tubular lumen (yellow arrow), blood congestion in the renal interstitium (blue arrow), swelling of hepatic cells with vacuoles, and moderate osteoporosis in the cytoplasm. The PTF group showed mild expansion of hepatic sinuses and splenic germinal center diffusion, and the renal tissues showed mild edema of epithelial cells accompanied by a large number of inflammatory cell infiltrations in the interstitial (red arrow). The PTF-SLNs group showed that the above symptoms improved, and there was only a slight inflammation in the hepatorenal tissues. The B-SLNs group showed that the nanocarriers did not damage the viscera. The results indicated that SLN loaded with PTF will not cause damage to the liver, kidney, and spleen but rather improve the performance of PTF to a certain extent.

## 3. Materials and Methods

### 3.1. Plant Material

*Phyllanthus emblica* L. was purchased from the Beijing Tibetan Hospital. The plant was collected in Nepal, identified by Prof. Chun-sheng Liu from the School of Chinese Materia Medica, Beijing University of Chinese Medicine, and deposited in the herbarium of Beijing University of Chinese Medicine. The PTF was extracted and purified by our team [12].

### 3.2. Materials and Reagents

Glyceryl monostearate (GMS, CAS number 123-94-4) and gallic acid (GA, CAS number 149-91-7) were purchased from Yuanye Bio-Technology Co., Ltd. (Shanghai, China). Lecithin (CAS number 93685-90-6), stearic acid (SA, CAS number 57-11-4), casein (CAS number 9000-71-9), and Tween-80 (CAS number 9005-65-6) were supplied by Macklin Bi-ochemical Co., Ltd. (Shanghai, China). Polyoxyethylene 20 cetylether (Brij^®^58, CAS number 9004-95-9) was obtained from Aladdin Biochemical Technology Co., Ltd. (Shanghai, China). Anhydrous sodium carbonate and phosphomolybdenum tungstic acid were provided by Guangfu Technology Development Co., Ltd. (Tianjin, China). Poloxamer 188 and sucrose were acquired from Fuchen Chemical Reagent Co., Ltd. (Tianjin, China).

### 3.3. Preparation of SLNs

PTF-SLNs were prepared by the thin film hydration method and then homogenized by probe sonication [30]. Briefly, the surfactant (10 mL 0.4% Brij^®^58) was dissolved in distilled water to form the aqueous phase, and the drug (10 mg PTF) was added. The solid lipid (3.75 mg GMS) and co-surfactant (30 mg lecithin) were dissolved in an appropriate amount of dichloromethane and placed in a 100 mL round-bottom flask to form the organic phase. The organic solvent was evaporated in a rotary evaporator at 40 °C and 45 rpm under vacuum until a thin film of dry lipid was obtained on the flask wall, and then left for some time to ensure the complete removal of the organic solvents [31]. Subsequently, the aqueous phase was added to this flask and sonicated to form a coarse emulsion. Last, the coarse emulsion was granulated by Ultrasonic Cell Crusher (JY92-ⅡN, Ningbo Scientz Biotechnology Co., Ltd., Ningbo, China) (4 s, 2 s, 240 W) for 5 min to form the mini emulsion with uniform particle size. Unincorporated PTF was further separated by ultra-centrifugal filters under 3000× *g* for 15 min at 4 °C at least three washing cycles. Taking particle size, PDI, zeta potential, entrapment efficiency (EE), and drug loading (DL) as indexes, the type of solid lipid (GMS, stearic acid, glyceryl behenate), rate of solid lipid to lecithin (1:4, 1:6, and 1:8), type and concentration of surfactant (0.1%, 0.2%, 0.4% tween-80, Brij^®^58, and poloxamer 188), the concentration of PTF (0.8 mg/mL, 1 mg/mL, and 1.2 mg/mL), cryoprotectant (glucose, sucrose, trehalose, glycine, mannitol, and *β*-cyclodextrin) were investigated to obtain the optimal formulation and preparation process. The blank SLNs (B-SLNs) were prepared in the same manner except for the absence of PTF.

### 3.4. HPLC Analysis

The chemical compositions of PTF and PTF-SLNs were determined by the Waters 2695 HPLC system equipped with a Waters 2996 photodiode array detector. The system control and data analysis were performed using Waters Empower 3 software. The chromatographic separation was performed on an Agilent Eclipse XDB-C18 (4.6 × 250 mm, 5 μL) using methanol (A) and 0.2% phosphoric acid aqueous solution (B) as the mobile phase. The mobile phase flow rate was kept at 0.5 mL/min, and the column temperature was maintained at 25 ℃. The gradient elution program was set as follows: 0–10 min, 5–15% A; 10–15 min, 15–25% A; 15–30 min, 25–30% A; 30–50 min, 30–60% A; 50–55 min, 60–90% A. The samples were detected at a wavelength of 270 nm, and the injection volume of each sample solution was 10 μL.

### 3.5. Detection of Total Phenolic and Tannin Content

UV scanning spectra of the PTF, PTF-SLNs, and B-SLNs were collected to detect the maximum absorption wavelength. A tungsten molybdophosphate-casein colorimetric method was used for the determination of the total phenolic content (TPC) and total tannin content (TTC) of PTF and PTF-SLNs with gallic acid as a reference substance, according to the method described by the Pharmacopoeia of China. The absorbance was measured at 760 nm by a UV-Vis Spectrophotometer (UV-2000, UNICO, Shanghai, China).

### 3.6. Characteristics of PTF-SLNs

PTF-SLNs was diluted with ultra-purified water to an appropriate concentration. The particle size, polydispersity index (PDI), and zeta potential of PTF-SLNs were measured using a Master-sizer apparatus (ZEN3600, Malvern Co., Malvern, PA, USA) (*n* = 3). In addition, pipette 50 μL diluted PTF-SLNs suspension placed on a copper grid coated with carbon and then negatively stained by applying one drop of aqueous phosphotungstic acid (1% *w*/*v*) solution. The excess staining solution was washed off the filter paper and allowed to dry at room temperature [32], then observed by a transmission electron microscope (TEM, Tecnai 12, Philips Co., Amsterdam, The Netherlands).

FTIR (ALPHA, Bruker Co., Zurich, Switzerland) was conducted to check the compatibility among the formulation’s components. Spectra of PTF, PTF-SLNs, and solid lipid (GMS) were taken in the 4000–400 cm^−1^ range. The peaks and patterns of PTF, PTF-SLNs, and GMS were recorded and compared.

The ultrafiltration method was used to determine the EE and DL [33]. In brief, a two milliliter PTF-SLNs suspension was transferred into a ten-milliliter brown volumetric flask, and then eight milliliter of methanol was added to this flask to dissolve PTF-SLNs. Subsequently, a two milliliter PTF-SLNs suspension was transferred into fifteen milliliters Millipore (UFC903096, 30 kDa, MWCO) and centrifuged on a high-speed freezing centrifuge (Sorvall ST 8R, Thermo Fisher, MA, USA) twice at 4 °C and 3000× *g* for 15 min each time. The unfiltered suspension was collected into a ten-milliliter brown volumetric flask and dissolved with eight milliliters of methanol. Lastly, the TPC before and after ultrafiltration was determined with the tungsten molybdophosphate-casein colorimetric method. The EE and DL were calculated according to the following formula: EE% = *W_a_*/*W_b_* × 100%; DL% = *W_a_*/(*W_b_* + *W_c_*) × 100%. (*W_a_*, the content of total phenols encapsulated in SLNs; *W_b_*, the initial content of total phenols; *W_c_*, the mass of carrier material).

The membrane dialysis method was used to evaluate the in vitro drug release. The dialysis membrane (molecular weight cut off 7000) that can retain PTF-SLNs and allow the PTF into the dissolution media was soaked in double distilled water for 12 h before use. One milliliter of PTF (4 mg/mL) solution and PTF-SLNs (containing equivalently 4 mg PTF) suspension were packaged in the membrane separately and placed in a thermostatic shaker at 37 °C and 100 rpm. Fifty milliliters of pH 5.0, 6.8, and 8.0 PBS was individually used as dissolution medium. A two milliliter release medium was pipetted into a twenty-five milliliter brown volumetric flask at fixed periodic times of 30 min, 1 h, 2 h, 4 h, 8 h, 12 h, 24 h, 36 h, 48 h, and 72 h for TTC determination with the tungsten molybdophosphate-casein colorimetric method. The cumulative release percentage was calculated with the following formula: $C_{c}=1/50\sum_{i=1}^{n=1}C_{d}$; $F_{t}=50\times \frac{C_{c}}{W_{t}}\times 100\%$. (*C_c_*, the corrected concentration; *F_t_*, the cumulative release percentage; *W_t_*, the total phenol content) (*n* = 3).

The stability study was implemented by preparing the optimized PTF-SLNs dispersion and lyophilization. The samples were kept at 4 °C and evaluated periodically for mean particle size and PDI. The appropriate amount of PTF-SLNs suspension was diluted in ultra-purified water. On the other hand, an appropriate amount of PTF-SLNs lyophilization dispersed in ultra-purified water, which continuously measured particle size and PDI for one week. Furthermore, in order to further investigate the stability of PTF-SLNs lyophilization, fifty milligram samples were accurately weighed into a twenty-five milliliters volumetric flask, which was dissolved and diluted to the scale of methanol. The TPC and TTC of PTF-SLNs lyophilization were periodically determined by UV-Vis spectrophotometry within one month. Three readings were taken for each sample to obtain mean  ±  SD.

### 3.7. In Vitro Study

Mouse Lewis lung cancer cells were obtained from the National Biomedical Cell Line Resource Center (Beijing, China) and were cultured in DMEM (Vicacell) supplemented with 10% (*v*/*v*) heat-inactivated fetal bovine serum (EXCell Bio) and 1% penicil-lin/streptomycin antibiotic (Gibco). In addition, the cells were kept at 37 °C in a humidified incubator with 5% CO_2_ and 95% air. Cells were plated in 100 cm^2^ flasks and allowed to grow to approximately 80–90% confluence before experimentation.

### 3.8. CCK8 Assay

The effect of PTF-SLNs on Lewis lung cancer cell viability was determined by CCK-8 assay and compared with PTF. The growing cells were seeded in 96-well plates at 5 × 103 cells/well. After a 24 h incubation period, the medium was removed and the cells were treated with 100 μL of medium containing various concentrations (2.19, 4.38, 8.75, 17.50, 35.00 μg/mL) of PTF-SLNs (calculated based on PTF content) and PTF for 48 h. Each concentration of formulations was repeated in five wells. Then, 10 μL CCK-8 solution (LABEAD, Beijing) was added to each well and incubated at 37 °C for 2–4 h. The absorbance was measured at 450 nm with a microplate well reader. The data were processed by Graph Pad Prism 9.

$$Cell\;inhibition\;rate=(A_{control}-A_{treat})/(A_{control}-A_{blank})$$

### 3.9. Apoptosis Analysis

The apoptosis was analyzed with flow cytometry using an annexin-V-fluorescein isothiocyanate (annexin-FITC) detection kit (KeyGEN Bio TECH Corp., Ltd., Jiangsu, China). Lewis lung cancer cells (2 × 105/well) were seeded in 6-well cell culture plates and treated with PTF and PTF-SLNs (equivalent to 20 μg/mL PTF) for 48 h. After the treatment, the medium was collected and cells were washed with PBS. Subsequently, the cells (1 × 106 cells/mL) were resuspended in 1 × binding buffer and stained using 5 µL each of AnnexinV-FITC and propidium iodide. The tubes were incubated at room temperature for exactly 15 min and protected from light. Finally, the cells were analyzed by flow cytometry and the data analysis was performed using Flow Jo 10 software.

### 3.10. Anti-Tumor Efficacy

Six-week male C57BL/6 mice were purchased from Beijing Vital River Laboratory Animal Technology Co., Ltd. All animal experiments complied with the Institutional Animal Care and Use Committee (approval code No. BUCM-4-2021061101-2055) of Beijing University of Chinese Medicine. C57BL/6 mice were inoculated subcutaneously in the right armpit with Lewis lung cancer cells (8 × 10^5^/mouse) suspended in PBS. When the tumor reached 50–100 mm^3^ in size, mice were divided into five groups (six mice per group).

The tumor-bearing mice were randomly grouped, which was the model group, 2 g/kg PTF free group, 0.4 g/kg PTF-SLNs group (calculated based on the actual content of PTF), and 0.4 g/kg B-SLNs group (*n* = 6). The mice were irrigation administrated once every day for 14 days. The 8 mg/kg cisplatin group as the positive drug group was administrated an intraperitoneal injection once within 14 days. Tumor volume and mice weight were recorded every other day, and mice were euthanized on day 14 after the treatment. The tumor volumes (mm^3^) were calculated by the following formula: $tumor\;volumes\left({mm}^{3}\right)=\left(L\times W^{2}\right)/2$, (*L*, the longest dimension; *W*, the dimension perpendicular to *L*). The tumor inhibition rate was calculated by the following formula: $tumor\;inhibitionrate\left(\%\right)=\left(W_{c}-{\;W}_{t}\right)/W_{c}\times 100\%$ (*W_c_*, the average tumor weight of the model group; *W_t_*, the average tumor weight of the treatment group).

### 3.11. Safety Preliminary Study

Mice were euthanized after treatment with different formulations, and blood was collected by enucleation of eyeballs. Tumor tissue and various organs, including heart, liver, spleen, lung, kidneys, stomach, and intestines were immediately harvested, isolated, and dried with tissue paper and weighed. They were fixed in 4% paraformaldehyde solution, embedded in paraffin, and stained with hematoxylin-eosin (H&E). The images were observed under a fluorescent inverted biological microscope (TS-2, Nikon, Tokyo, Japan). In addition, the hepatorenal function was evaluated by analyzing biochemical indicators of plasma such as AST, ALT, CREA, and UREA using the automatic biochemical analyzer (DxC700AU, Beckman Coulter, USB).

### 3.12. Statistical Analysis

Statistical analysis was performed using SPASS 20 and Prism GraphPad 8.3 software. The experimental data were expressed as the mean values ± standard deviation (SD). Statistical differences between groups were assessed using a one-way ANOVA. The differences were considered significant at the level of * *p* < 0.05, and the difference was extremely significant when ** *p* < 0.01.

## 4. Conclusions

Solid lipid nanoparticles have emerged as multipurpose nanomedicines for various drug delivery systems. PTF-SLNs have been successfully developed by the thin film hydration method to enhance therapeutic outcomes in lung cancer. In this research, we used biocompatible and biodegradable constituents to investigate the preparation and prescription process of SLNs and their anti-tumor ability. The optimal PTF-SLNs allowed high drug incorporation with significantly improved aqueous solubility of PTF. The characterization results of PTF-SLNs exhibited desirable physicochemical properties, including good physical stability with a small particle size and favorable control effect of in vitro drug release. The pharmacodynamics of in vitro and in vivo studies revealed that PTF-SLNs exhibited a stronger anti-tumor effect than PTF. Despite decreasing the dosage of PTF, PTF-SLNs still displayed robust anti-tumor effects. The calculated IC50 values for the PTF and PTF-SLNs were 67.43 μg/mL and 20.74 μg/mL, respectively. In addition, the tumor inhibition rates of 2 g/kg PTF and 0.4 g/kg PTF-SLNs were 59.97% and 64.55%, respectively. The safety preliminary study confirmed that the PTF-SLNs are safe and non-toxic. It can be concluded that by administering PTF-SLNs via the oral route, the anticancer potential of PTF was improved without any damage, indicating the developed PTF-SLNs’ ability to reduce the dose-related side effects of PTF. Thus, SLNs may be a promising approach for PTF oral supplementation, possibly leading to the enhancement of PTF anti-tumor abilities. This finding is promising for herbal delivery This finding is promising for PTF applied in the clinical treatment of lung cancer and SLNs widely applied in the herbal delivery system.

## Figures and Tables

**Table 1:** Screening of nanocarrier based on entrapment efficiency (EE), drug loading (DL), particle size, and polydispersity index (PDI).

Formulation Variables	% EE	% DL	Size/nm	PDI
SA	61.38 ± 0.78	5.10 ± 1.47	42.44 ± 1.73	0.228 ± 0.26
GMS	59.78 ± 2.31	5.57 ± 1.03	47.76 ± 0.97	0.220 ± 0.44
GB	53.67 ± 3.53	4.69 ± 1.92	73.66 ± 2.23	0.315 ± 0.52
tween-80	48.96 ± 2.86	2.70 ± 0.49	79.14 ± 3.18	0.356 ± 0.10
Brij^®^58	67.42 ± 1.04	2.48 ± 0.81	71.12 ± 0.66	0.393 ± 0.05
Poloxamer 188	39.17 ± 2.55	1.94 ± 0.58	56.81 ± 1.21	0.385 ± 0.21

**Table 2:** Single factor analysis of the PTF-SLNs preparation.

PTF/mg	GMS/Lecithin	Brij^®^58/%	% EE	% DL	Size/nm	PDI
8	1:6	0.4	71.87 ± 0.89	3.88 ± 2.71	46.77 ± 0.91	0.289 ± 1.73
8	1:8	0.2	65.22 ± 3.21	4.88 ± 2.97	40.99 ± 2.36	0.183 ± 1.26
8	1:4	0.1	55.58 ± 0.93	3.88 ± 1.08	50.17 ± 2.73	0.235 ± 1.92
10	1:8	0.4	75.38 ± 1.03	5.02 ± 0.41	41.29 ± 1.58	0.181 ± 2.51
10	1:6	0.1	60.50 ± 1.13	4.96 ± 0.50	45.27 ± 0.08	0.178 ± 5.72
10	1:4	0.2	57.09 ± 2.37	3.94 ± 1.14	44.77 ± 2.29	0.179 ± 1.19
12	1:8	0.1	47.22 ± 2.76	3.61 ± 2.11	45.04 ± 2.22	0.158 ± 0.56
12	1:4	0.4	46.68 ± 3.89	2.72 ± 0.97	43.96 ± 3.10	0.170 ± 2.35
12	1:6	0.2	55.99 ± 1.62	3.96 ± 0.44	45.49 ± 1.18	0.130 ± 2.39

**Table 3:** The effect of cryoprotectants on PTF-SLNs.

Lyophilized Protective Agent	Appearance	Re-Dispersibility	Particle Size (nm)	PDI
No	Collapsed	Poor	-	-
4% glucose	Collapsed	Good	156.12 ± 1.06	0.234 ± 0.42
6% glucose	Delamination, partial collapse	Good	135.74 ± 1.31	0.526 ± 0.77
8% glucose	Delamination, partial collapse	Good	162.00 ± 3.56	0.509 ± 1.20
4% sucrose	Even and full	Good	132.14 ± 2.33	0.259 ± 2.90
6% sucrose	Even and full	Good	118.73 ± 5.11	0.211 ± 0.72
8% sucrose	Even and full	Good	118.91 ± 1.32	0.204 ± 1.35
4% glycine	Collapsed	Good	161.85 ± 3.83	0.244 ± 1.43
6% glycine	Collapsed	Good	126.04 ± 0.99	0.399 ± 2.88
8% glycine	Collapsed	Good	201.67 ± 3.60	0.497 ± 1.94
4% mannitol	Delamination, partial collapse	Good	171.63 ± 0.63	0.495 ± 0.80
6% mannitol	Delamination, partial collapse	Good	161.33 ± 1.41	0.486 ± 1.22
8% mannitol	Delamination, partial collapse	Good	128.28 ± 1.05	0.461 ± 0.45
4% *β*-cyclodextrin	Delamination, partial collapse	Good	106.87 ± 0.02	0.332 ± 0.30
6% *β*-cyclodextrin	Even and full	Good	121.42 ± 2.18	0.414 ± 2.19
8% *β*-cyclodextrin	Even and full	Good	123.09 ± 2.55	0.971 ± 1.72

**Table 4:** The effect of tumor growth inhibits each group (*n* = 6).

Groups	Body Weight (g)	Tumor Volume (mm^3^)	Tumor Weight (g)	TGI (%)
Model	23.11 ± 1.09	1219 ± 398	1.29 ± 0.73	-
0.4 mg/kg B-SLNs	23.17 ± 0.5	918 ± 579	0.73 ± 0.17	20.38
8 mg/kg Cisplatin	22.54 ± 1.05	878 ± 369	0.51 ± 0.06	42.82
2 g/kg PTF	18.73 ± 1.06	525 ± 91	0.46 ± 0.14	59.97
0.4 g/kg PTF-SLNs	19.2 ± 1.73	460 ± 88	1.02 ± 0.72	64.55

## Data Availability

Not applicable.
